# Phytochemistry, bioactivity, and medicinal use of *Cannabis sativa* roots: a comprehensive review

**DOI:** 10.1186/s42238-026-00470-4

**Published:** 2026-07-21

**Authors:** Tiago Antônio Heringer, Chana de Medeiros da Silva, Daniela Fernandes Ramos, Lia Gonçalves Possuelo

**Affiliations:** 1 University of Santa Cruz Do Sul, Santa Cruz Do Sul, Brazil; 2 Federal University of Rio Grande – FURG, Rio Grande, Brazil

**Keywords:** Cannabis, Medical, Plants, Medicinal, Phytochemicals, Plant Roots

## Abstract

**Background:**

Cannabis sativa is among the earliest domesticated plants and has been widely utilized for nutritional, industrial, and medicinal purposes. While its aerial parts, particularly the leaves and inflorescences, have been extensively studied due to their recreational and therapeutic applications, the roots remain comparatively neglected, despite their long-standing use in traditional medicine for treating wounds, burns, and inflammation.

**Aim:**

This review aims to summarize the current knowledge on the phytochemical composition and biological activities of C. sativa roots, with emphasis on their pharmacological potential.

**Results:**

The roots of C. sativa contain a diverse spectrum of secondary metabolites, including phytosterols, alkaloids, terpenes, and phenolic compounds. These bioactive constituents have been reported to exert antioxidant, anti-inflammatory, and antimicrobial activities. In vivo studies further demonstrate nociceptive and antispasmodic effects, with no evidence of cytotoxicity.

**Conclusion:**

C. sativa roots represent an underexplored pharmacognostic resource with significant potential for the discovery of novel bioactive compounds. Their chemical diversity and biological activities provide a strong rationale for renewed scientific attention, supporting future research into this underestimated part of a species of growing medical and economic relevance.

## Introduction

*Cannabis sativa* was originally domesticated in Eurasia around 2737 BC and was one of the first cultivated plants. Its fibers are widely used to make fabrics, the leaves are used to feed animals, and the seeds are consumed by humans, in addition to the psychoactive use of its flowers (Araújo et al. 2024).

Although currently cultivated on a global scale, this plant exhibits distinct phenotypic variations that are directly influenced by the specific geographical environment in which it develops (Odieka et al. 2022). Despite ongoing taxonomic debates, the prevailing convention classifies the genus *Cannabis* as a single species, *Cannabis sativa* L. (Schilling et al. 2020). Since antiquity, this species has been recognized for its therapeutic properties, being extensively utilized in both traditional and modern medicine due to the rich diversity of phytochemicals biosynthesized across various parts of its botanical structure (Ryz et al. 2017).

More than 500 compounds have been identified and most of them are cannabinoids, which were first discovered in this species. Notably, cultivars of this plant also hold other classes of secondary metabolites, such as phenols, flavonoids, alkaloids, terpenes, steroids, and resins (Dalli et al. 2023).

As a medicinal plant, *C. sativa* roots were initially used to relieve pain, heal burns, and treat infected wounds. However, the leaves and inflorescences became the primary focus of scientific inquiry due to the extensive clinical research into the therapeutic properties and pharmacological activity of the cannabinoids concentrated in the plant’s aerial parts (Hurgobin et al. 2021). In this context, the roots have been the subject of several studies on their phytochemical composition, but few studies have addressed their medicinal potential (Kornpointner et al. 2021).

This study aimed to conduct a review to identify the bioactive compounds and biological activities described for *C. sativa* roots.

## Methods

Searches were conducted in the following databases: Index Medicus Western Pacific (WPRO), Latin American and Caribbean Health Sciences Literature (LILACS), Spanish Bibliographic Index of Health Sciences (IBECS), and SCOPUS, as well as PubMed, Cochrane Library, Web of Science, and Scientific Electronic Library Online (SciELO). The keywords used were: “roots” AND “*Cannabis sativa*” AND (“extract” OR “medicinal use”).

Studies published up to December 2024 were included, with no limit on publishing date. Firstly, evaluators checked the titles and abstracts, looking for words that referred to tests for the medicinal use of the roots. Full papers in Portuguese and English were included if they had conducted in vitro and in vivo analyses using *C. sativa* roots extracts for medicinal use or compounds isolated from these extracts. Review articles, duplicate studies, and paid access papers were excluded.

### Bioactive compounds

Plants produce various compounds to protect them from microorganisms and other environmental factors. When consumed, these compounds alleviate oxidative stress, inflammatory processes, and prevent various diseases (Moges et al. 2025). Table 1 shows the compounds identified in *C. sativa* roots, their isolated amounts, and their biological activities, which will be divided and discussed according to their classes.

**Table 1:** Biological activities of compounds found in *C. sativa* roots

Class	Compound	Quantities(^ref^)	Biological Activity	Reference
**Phenolic compounds**				
	Vanillin	0,0076 mg/g (Elhendawy et al. 2019) 0,0006 mg/g (Huang et al. 2023)	Antitumor, neuroprotective, antimicrobial	(Arya et al. 2021; Elhendawy et al. 2019; Huang et al. 2023)
	*p*-Coumaroyltyramine	0,01978 mg/g (Elhendawy et al. 2019)	Antitumor, antibacterial, antiurease, antifungal, antiviral, neuroprotective and cardioprotective	(Araújo et al. 2024; Bakrim et al. 2024; Elhendawy et al. 2019; Menezes et al. 2021)
	Serinic acid	0,0011 mg/g (Huang et al. 2023)	Antioxidant and anti-inflammatory	(Bartel et al. 2024; Huang et al. 2023)
	Cetopinoresinol	0,00335 mg/g (Huang et al. 2023)	Antitumor, antioxidant and immunomodulatory	(Chen et al. 2012; Huang et al. 2023)
	Feruloyltyramine	0,00445 mg/g (Huang et al. 2023)	Anti-inflammatory, anti-asthmatic	(Araújo et al. 2024; Menezes et al. 2021; Gagné et al. 2024)
	Dihydrobenzofuran	0,0006 mg/g (Huang et al. 2023)	Antiparasitic	(Judžentienė et al. 2023; Omar et al. 2016)
	Vanillic acid	0,00625 mg/g (Huang et al. 2023)	Anti-inflammatory	(Huang et al. 2023)
	Syringol	< LOQ (Huang et al. 2023)	Anti-tumor	(Huang et al. 2023; Intisar et al. 2012)
**Alkaloids**				
	Cannabisativin	< LOQ (Araújo et al. 2024)	Analgesic, antiparasitic, antipyretic, antiemetic, antitumor, diuretic and analgesic	(Araújo et al. 2024; Lowe et al. 2021; Huang et al. 2023; Menezes et al. 2021; Gagné et al. 2024)
**Cannabinoids**				
	Cannabichromene		Anti-inflammatory and anti-tumor	(Maiocchi et al. 2024; Whynot et al. 2023; Huang et al. 2023)
	Cannabinol	0,0002 mg/g (Huang et al. 2023)	Antimicrobial, anti-inflammatory, sleep regulator	(Huang et al. 2023; Saleska et al. 2024; Gu et al. 2019)
	Cannabivarin	0,00045 mg/g (Huang et al. 2023)	Antitumor	(Whynot et al. 2023; Huang et al. 2023)
		0,0003 mg/g (Huang et al. 2023)	Neurological effects, rheumatological pain, sleep regulator and anxiety disorders	(Carvalho et al. 2024; Komatsu et al. 2024; Pathak et al. 2024; Wang et al. 2024; Huang et al. 2023; Saleska et al. 2024)
	Cannabidiolic acid	< LOQ (Gagné et al. 2024)	Antimicrobial, antinausea and antitumor effects	(Gagné et al. 2024; Pellati et al. 2018)
**Terpenes**				
	Glutinol	< LOQ (Kornpointner et al. 2021)	Antitumor and antidiabetic	(Kornpointner et al. 2021; Fantoukh et al. 2023; Abdel-Sattar et al. 2015; Pamunuwa et al. 2016)
	Friedelanol	0,239 mg/g (Ferrini et al. 2022) 0,06–0,20 mg/g (Kornpointner et al. 2021)	Antitumor, anti-inflammatory and antioxidant	(Kornpointner et al. 2021; Oliveira et al. 2024; Waghole et al. 2022; Elhendawy et al. 2019; Huang et al. 2023; Ferrini et al. 2022)
	_Friedelin_	0,42—0,71 mg/g (Kornpointner et al. 2021) 0,288 mg/g (Ferrini et al. 2022) 0,065 mg/g (Huang et al. 2023)	Antitumor, anti-inflammatory, antimicrobial and antifungal	(Kornpointner et al. 2021; Singh et al. 2023; Elhendawy et al. 2019; Huang et al. 2023; Judžentienė et al. 2023; Ferrini et al. 2022)
	β-Amyrin	< LOQ (Kornpointner et al. 2021)	Anti-inflammatory, analgesic, antihyperglycemic and nephroprotective	(Judžentienė et al. 2023; Alam et al. 2024; Angsusing et al. 2024; Roohi et al. 2024)
	β-Amirone	< LOQ (Kornpointner et al. 2021)	Analgesic, antidiarrheal and hypoglycemic	(Kornpointner et al. 2021; Taher et al. 2024)
	Oleanolic acid	0,0014 mg/g (Huang et al. 2023)	Antitumor, antimycobacterial	(Zhou et al. 2024; Huang et al. 2023; Deng et al. 2024; Mazzarotti et al. 2024; Saini et al. 2024)
	Ursolic acid	< LOQ (Gagné et al. 2024)	Antitumor, antimycobacterial	(Gagné et al. 2024; Saini et al. 2024; Zeng et al. 2024)
**Phytosterols**				
	Fucosterol	< LOQ (Kornpointner et al. 2021)	Antiviral, antitumor, antiparasitic	(Kornpointner et al. 2021; Li et al. 2021; Mo et al. 2018; Perumal et al. 2018)
	_Oleamide_	< LOQ (Kornpointner et al. 2021)	Antibacterial and sleep regulation	(Kornpointner et al. 2021; Purwantiningsih et al. 2024; Roy et al. 2021)
	Ethyl palmitate	< LOQ (Kornpointner et al. 2021)	Antiviral and anti-inflammatory	(Kornpointner et al. 2021; Sagna et al. 2023; Saeed et al. 2012)
	_Ethyl linoleate_	< LOQ (Kornpointner et al. 2021)	Healing, antimicrobial, antioxidant and anti- inflammatory	(Kornpointner et al. 2021; Alavi and Ashengroph 2023; Park et al. 2014; Jelenko et al. 1975)
	Ethyl stearate	< LOQ (Kornpointner et al. 2021)	Anti-inflammatory and neuroprotective	(Kornpointner et al. 2021; Huang et al. 2023; Miya et al. 2023)
	_β-Sitosterol_	0,687 mg/g (Ferrini et al. 2022)	Antiviral, antitumor, analgesic and antidiarrheal	(Kornpointner et al. 2021; Khan et al. 2022; Elhendawy et al. 2019; Huang et al. 2023; Ferrini et al. 2022; Taher et al. 2024)
	Campesterol	0,172 mg/g (Ferrini et al. 2022)	Antimicrobial	(Freitas da Silva et al. 2023; Elhendawy et al. 2019; Judžentienė et al. 2023; Ferrini et al. 2022)
	Stigmasterol	0,202 mg/g (Ferrini et al. 2022)	Anti-inflammatory	(Yu et al. 2022; Judžentienė et al. 2023; Ferrini et al. 2022; Angsusing et al. 2024)
	Palmitic acid	< LOQ (Judžentienė et al. 2023)	Anti-tumor	(Xu et al. 2024; Judžentienė et al. 2023; Zhu et al. 2021)
	γ-Sitosterol	< LOQ63	Antiviral and antitumor	(Shokry et al. 2023; Khan et al. 2022; Judžentienė et al. 2023)
	Palmitoleic acid	0,03185 mg/g (Elhendawy et al. 2019)	Antibacterial	(Elhendawy et al. 2019; Han et al. 2024; Xu et al. 2025)
	Ergosterol peroxide	0,0007 mg/g (Huang et al. 2023)	Antiviral and antimycobacterial	(Zhou et al. 2019; Chiou et al. 2024; Duan et al. 2021; Duarte et al. 2007; Huang et al. 2023)
	α-linolenic acid	0,00255 mg/g (Huang et al. 2023)	Antitumor, anti-inflammatory and antioxidant	(Yuan et al. 2022; Huang et al. 2023)
	Linoleic acid	0,00125 mg/g (Huang et al. 2023)	Antioxidant and anti-tumor	(Huang et al. 2023; Gagné et al. 2024)
	Dotriacontene	0,0007 mg/g (Huang et al. 2023)	Anti-inflammatory	(Huang et al. 2023)
	Pentadecanoic acid	0,00885 mg/g (Elhendawy et al. 2019)	Antibiofilm, anti-inflammatory, antifibrotic and antitumor	(Galdiero et al. 2021; Venn-Watson and Schork 2023; Huang et al. 2023)
	Palmitoleic acid	0,03185 mg/g (Elhendawy et al. 2019)	Anti-inflammatory and antimicrobial	(Huang et al. 2023; Tabashsum et al. 2025)
	Stearolic acid	< LOQ (Gagné et al. 2024)	Anti-parasitic	(Gagné et al. 2024; Alloatti et al. 2013; Londero et al. 2018)

Caption: < LOQ—Below Limit of Quantification; (^ref^)—Reporting study

### Phenolic compounds

Phenolic compounds are a heterogeneous group of phytochemicals found in plants, divided into classes according to their chemical structure and biological function. These metabolites are crucial for the defense mechanisms of the plant and can contribute to human health with its anti-inflammatory, antioxidant, and antimicrobial properties (Baptista et al. 2024). Phenolic compounds can be divided into groups such as phenolic acids, flavonoids, tannins, and lignans, which have a basic structure containing an aromatic ring with one or more hydroxyls. Their antioxidant capacity can be closely related to the number of free hydroxyls; thus compounds that donate more electrons have a higher antioxidant activity (de Castro et al. 2022).

One of the phenolic compounds isolated from *C. sativa* roots was vanillin, a substance widely used in the industry as a food flavoring. A range of therapeutic properties have been associated to it, such as antitumor, neuroprotective, and antimicrobial activity, as well as being used to potentiate the action of antibiotics such as gentamicin, chloramphenicol, ciprofloxacin, and meropenen against resistant strains of *P. aeruginosa, S. aureus*, and *E. coli (*Arya et al. 2021).

*P*-coumaroyltyramine helps to prevent heart and vascular diseases and neurological disorders, and is also effective in antitumor, antibacterial, antiurease, antifungal, and antiviral activity tests (Bakrim et al. 2024). Serinic acid works as a nutraceutical, improving oxidative damage, anti-inflammatory action, thus reducing metabolic risks and dyslipidemia (Bartel et al. 2024). Cetopinoresinol has anti-tumor activity due to its immunomodulatory and antioxidant capacity (Chen et al. 2012).

### Alkaloids

Alkaloids are nitrogen-based organic compounds that have various biological activities. Due to presenting different chemical structures, they can be divided into isoquinolines, pyridines, quinolines, among others. Such composition enables various actions and effects such as anticancer, anti-inflammatory, antibacterial, and antiviral activities (Chua et al. 2024; Yan et al. 2021). These organic compounds have at least one nitrogen atom in an amine-type structure, which is usually incorporated into a cyclic system. This characteristic allows them to react with acids to form salts (Eguchi et al. 2019).

Cannabisativine is an alkaloid that was first isolated from *C. sativa* roots in 1975. By investigating this compound, a study has found analgesic and antiparasitic activities, as well as antipyretic, antiemetic, antitumor, diuretic, and analgesic capabilities (Lowe et al. 2021). It is a macrolactam, which is a cyclic amide derived from carboxylic amino acids and characterized by a 1-azacycloalkan-2-one structure (or analogues) with unsaturation or heteroatoms replacing one or more carbon atoms of the ring. Its chemical structure includes three fused rings: a pyridine ring, a piperidine ring, and a 13-membered lactam ring, and it has hydroxyl and amine functional groups (Radwan et al. 2021).

### Cannabinoids

This class is not found in large quantities in roots. They have a carbocyclic structure with 21 carbon atoms, generally formed by three rings: cyclopentane, tetrahydropyran, and benzene. The main cannabinoids include Δ⁹-tetrahydrocannabinol (Δ⁹-THC), cannabidiol (CBD), and cannabinol (CBN) (Barrales-Cureño et al. 2020).

CBD is the main cannabinoid found in *C. sativa* and is the most studied one, known for its neurological effects, aid in pain alleviation, and treatment of rheumatological diseases and disorders such as anxiety (Carvalho et al. 2024; Komatsu et al. 2024; Pathak et al. 2024; Wang et al. 2024). Another cannabinoid found in the roots is tetrahydrocannabivarin, which is very similar in nomenclature to Δ⁹-THC. Notably, it has very different characteristics, having been tested as an appetite regulator for the treatment of obesity and diabetes (Haghdoost et al. 2024). Cannabichromene has anti-inflammatory activity (Maiocchi et al. 2024) and shows results in reducing the proliferation of bladder cancer cells, along with other cannabinoids such as cannabivarin (Whynot et al. 2023).

### Terpenes

These are hydrocarbons derived from isoprene (C₅H₈) and can be classified according to the number of isoprene units they contain: monoterpenes (C₁₀H₁₆), sesquiterpenes (C₁₅H₂₄), diterpenes (C₂₀H₃₂), among others. Their basic structure is made of multiple isoprene units joined head-to-tail, forming linear chains or complex cyclic structures that have antioxidant, anti-tumor, and anti-inflammatory characteristics (Lukman et al. 2024).

Glutinol is one of the compounds found in *C. sativa* roots, which has shown antitumor action against MCF-7 breast tumor cell lines (Fantoukh et al. 2023). Some compounds derived from this terpene have shown antifungal activity against phytopathogenic fungi of the genus *Fusarium *(2024). Also, friedelanol has antitumor and anti-inflammatory properties (Oliveira et al. 2024; Waghole et al. 2022). Friedelin is another triterpene of the same class isolated from roots, presenting antitumor and anti-inflammatory properties, as well as antimicrobial activity against: Gram-positive bacteria, such as *S. aureus, S. epidermidis,* and *Bacillus cereus*; Gram-negative bacteria, such as *Proteus mirabilis, E. coli, K. pneumoniae*; and the fungi *Aspergillus fumigates* and *Trichophyton rubrum *(Singh et al. 2023).

### Phytosterols

Phytosterols are plant sterols structurally similar to cholesterol, characterized by a tetracyclic cyclopentanoperhydrophenanthrene nucleus with a side chain on carbon 17. The most common phytosterols include β-sitosterol, campesterol, and stigmasterol, which have a double bond at position 5 and methyl or ethyl substituents on the side chain at carbon 24 (Barkas et al. 2023). Phytosterols were the most found class in the studies included in this review. They are nonpolar substances found in different plant species and studied for various purposes due to their high antioxidant capacity (Liu et al. 2024). β and γ-sitosterol are esters analogous to cholesterol, which have antiviral properties (Shokry et al. 2023) and antitumor activities (Khan et al. 2022), which are also found in fucosterol (Jiang et al. 2023; Li et al. 2021; Mo et al. 2018), and antiparasitic properties when tested against *Plasmodium falciparum (*Perumal et al. 2018).

Oleamide has demonstrated antibacterial properties against the bacterium *P. aeruginosa (*Purwantiningsih et al. 2024), and is an important endocannabinoid in sleep regulation (Roy et al. 2021). Ethyl palmitate has antiviral (Sagna et al. 2023) and anti-inflammatory properties (Saeed et al. 2012), whereas ethyl linoleate is effective as a wound healer, antimicrobial, antioxidant (Alavi and Ashengroph 2023), and anti-inflammatory (Park et al. 2014). Campesterol has emerged as an alternative source of new antimicrobial substances and has been tested against various bacteria with semi-synthesized compounds from campesterol (Freitas da Silva et al. 2023).

Studies have indicated antitumor activity of palmitic acid (Zhou et al. 2024; Xu et al. 2024), Ergosterol peroxide has promising antiviral properties against different viruses (Zhou et al. 2019; Chiou et al. 2024; Duan et al. 2021) and is active in tests against MTB (Duarte et al. 2007), while α-linolenic acid has antitumor, anti-inflammatory, and antioxidant activities (Yuan et al. 2022). Pentadecanoic acid has shown antibiofilm activity against *C. albicans* and *K. pneumoniae (*Galdiero et al. 2021), anti-inflammatory, antifibrotic, and anticancer activities via the activation of enzymes such as Adenosine Monophosphate-Activated Protein Kinase (AMPK), and inhibition of the JAK-STAT pathway, which signals cytokines that control the inflammatory process (Venn-Watson and Schork 2023).

Different extraction processes were conducted: cold maceration; hot and cold maceration; fermentation and cold maceration; hot maceration with fermentation, and cold maceration. By using these different processes, authors aimed to test the increased effectiveness in obtaining compounds and the antioxidant capacity of each one, proving the effectiveness of obtaining stigmasterol according to the increased complexity of the extraction process (Yu et al. 2022).

### Biological activities

The use of *C. sativa* roots in traditional medicine has led to some research using extracts of this pharmacogen, and tests have been conducted on its antimicrobial, anti- inflammatory, and antioxidant activity in vitro, as well as evaluating other biological activities in vivo, such as antipyretic, antinociceptive, and antispasmodic action.

### Antimicrobial activity

The antimicrobial activities of the isolated compounds of the extracts were tested focusing on their medicinal use (Table 2). Two compounds showed promising activity: campesterol and *p*-coumaroyltyramine; The first one was found to hold fungicidal activity against C. neoformans with an IC50 value of 13.7 μg/mL. The latter one showed activity against the Gram-negative bacterium E. coli with an IC50 value of 0.8 μg/mL (Elhendawy et al. 2019). Campesterol is a phytosteroid present in different plant species, some of which have anti-inflammatory activity (Wang et al. 2020), and *p*-coumaroyltyramine is known for its antiviral, antimicrobial, and anticoagulant activities (Masi et al. 2021). The extracts had inhibitory results when tested against *S. aureus, E. coli,* and *C. albicans.* Note that the concentration used to inhibit Gram-positive bacteria was high (> 800 µg/mL) (Gagné et al. 2024).

**Table 2:** Antimicrobial results of studies with *Cannabis sativa* roots

Compound	Microorganism	MIC	ref
Campesterol^1^	*C. neoformans*	13.7 μg/mL	Elhendawy et al. 2019)
p-coumaroyltyramine^1^	*E. coli*	0.8 μg/mL	
AgNPs	*S. aureus*	65 μg/mL	Suman et al. 2022)
	*E. coli*	70 μg/mL	
	*P. aeruginosa*	75 μg/mL	
	*K. pneumoniae*	60 μg/mL	
Aqueous extract^2^ Acid–base extract^2^	*C. albicans*	180 μg/mL 120 μg/mL	Gagné et al. 2024)

Caption: ^1^—Isolated compound; ^2^—Extract

*AgNPs* silver nanoparticles, *MIC* minimum inhibitory concentration

Another study tested the antimicrobial capacity of Cannabis roots by obtaining silver nanoparticles (AgNPs). The ethanolic extract was used to obtain the AgNPs, which were tested against Gram-positive and Gram-negative bacteria, obtaining the following minimum inhibitory concentrations: *S. aureus* (65 μg/mL), *E. coli* (70 μg/mL), *P. aeruginosa* (75 μg/mL), and *K. pneumoniae* (60 μg/mL)91. This same study tested the hemolytic capacity and cytotoxic effect of AgNPs, founding no effect. The extract was not characterized. Nevertheless, obtaining and analyzing the AgNPs was promising, because they have emerged as a new alternative treatment for resistant bacteria, acting mainly on the membranes of microorganisms, facilitating the permeability of other compounds or changing the physicochemical properties of this membrane by breaking it (Alavi and Ashengroph 2023).

### Anti-inflammatory properties

The anti-inflammatory capacity of the *Cannabis* root extract and the isolated compounds, two triterpenes and one phytosterol, showed promising results in both the extract and the terpenic compounds, helping to prevent the expression of inflammation markers such as interleukin IL6 and Tnfα (Ferrini et al. 2022). When measuring the production of IL-6 by liposaccharide stimulation of differentiated human monocytic leukemia (THP-1) cells, a significant result was obtained from the aqueous extract of *C. sativa* roots (Gagné et al. 2024).

In another study, the anti-inflammatory properties of the isolated compounds were tested, in which the six isolated cannabinoids showed anti-inflammatory effects by reducing pro-inflammatory cytokines in an inflammasome model with THP-1 cells. Six of the other non-cannabinoid compounds reduced the expression of the IL1β and TNFα markers (Huang et al. 2023). In recent years, the anti-inflammatory and immunomodulatory capacity of terpenes has been widely studied. Notably, they are abundant in various plant species, so they have been increasingly highlighted as anti-inflammatory and immunomodulatory compounds (Miranda et al. 2022). Phytosterols are similar in structure to cholesterol and have antitumor, antioxidant, and anti-inflammatory properties (Liu et al. 2022). Phenols and polyphenols can chelate or annul oxidizing compounds, acting as natural anti-inflammatories (Fernandes et al. 2023).

A study evaluated the anti-inflammatory effect of the cannabis roots extracts using RAW264.7 murine macrophage cells, which were treated with liposaccharides. Besides evaluating the anti-inflammatory effect, the authors also investigated the expression of the Nitric Oxide Synthesis Inducing Protein (iNOS), an inflammatory marker. The authors identified that the anti-inflammatory effect was proportional to the complexity of the extraction and the amount of stigmasterol in the sample (Yu et al. 2024).

### Antioxidant properties

The antioxidant activity of the root extract (Table 3) and of the isolated compounds in which the extract performed best (80%), with most compounds being slightly less efficient (65%), was in the 2,2-diphenyl-1-picrylhydrazyl radical (DPPH) test. There was a difference in the chelating capacity between the isolated compounds showing the extract was the most efficient (80%), followed by β-sitosterol (75%), in the Fe + chelating test. The same results were obtained when tested in cell culture to see if the antioxidant capacity was maintained. Note that when the cytotoxicity and DNA damage capacity of *C. sativa* root extracts were tested, no damage to DNA structures was ideintified (Ferrini et al. 2022).

**Table 3:** Antioxidant analysis of studies on *Cannabis sativa* roots

Extract/compound	Test performed	Results	Ref
Friedelin^1^	DPPH Fe chelator	832.4 ± 1.7 μg/mL 883.5 ± 7.2 μg/mL	Ferrini et al. 2022)
β-Sitosterol^1^	DPPH Fe chelator	920.2 ± 3.4 μg/mL 858.8 ± 6.2 μg/mL	
Friedelanol^1^	DPPH Fe chelator	875.1 ± 1.7 μg/mL 547.6 ± 6.3 μg/mL	
Ethanolic^2^	DPPH Fe chelator	420.1 ± 2.1 μg/mL 385.5 ± 3.0 μg/mL	
Ethanolic^2^	FRAP ABTS Intracellular oxidation	97.1 ± 3.8 89.1 ± 2.5 0.809 ± 0.013	Kornpointner et al. 2021)
Methanolic^2^	DPPH	1.556 ± 0.004 mmol/L	Judžentienė et al. 2023)

Caption: ^1^—Isolated compound; ^2^—Extract

*DPPH* 2,2-diphenyl-1-picrylhydrazyl radical, *FRAP* Iron Antioxidant Power, *ABTS* antioxidant capacity of 2,2'- azinobis-3-ethylbenzothiazoline-6-sulfonic acid

Another study tested antioxidant capacity using the Iron Antioxidant Power (FRAP) and 2,2'-azinobis-3-ethylbenzothiazoline-6-sulfonic acid (ABTS) techniques. The researcher also employed an in vivo technique, using the fungus Saccharomyces cerevisiae to assess the intracellular antioxidant power, obtaining a promising result. However, this capacity cannot be attributed to the triterpenes, since another sample with more triterpenes showed the lowest antioxidant action value, which may be due to the phytosterols (Kornpointner et al. 2021). When the antioxidant capacity was tested using the ABTS test, the aqueous extract showed greater antioxidant capacity when compared to the ethanolic and basic acid extracts (Gagné et al. 2024). An analysis of the antioxidant potential by hydrogen peroxide inhibition (H_2_O_2_) was conducted, in which the roots showed antioxidant capacity (Judžentienė et al. 2023). Secondary metabolites have a high antioxidant capacity and the main ones include phenolic compounds, terpenes, and alkaloids (Oreopoulou et al. 2021).

### Biological activities in vivo

A study tested the extracts as antipyretics in young Rattus norvegicus males, in addition to testing the antinociceptive, anticonvulsant, and antispasmodic capacity in Mus musculus specimens. The aqueous extract of the roots was able to reduce contortions caused by acetic acid in *M. musculus* at a concentration of 12.5 mg/kg and did not have the same result at higher concentrations. A similar result was observed when the antinociceptive capacity was tested, in which a reduction in pain was observed at a concentration of 12.5 mg/kg. In the hot plate pain latency test, the rodents had an increase in pain latency at all concentrations of the extract (12.5, 25 and 50 mg/kg). No antipyretic or spasmolytic activity was observed with this extract at any of the concentrations (Menezes et al. 2021).

Also in murine models, the action of the aqueous extract of *C. sativa* roots was used to attempt the reduction of abdominal contractions caused by primary dysmenorrhea, which was effective at concentrations of 12.5, 25 and 50 mg/kg, demonstrating an antidysmenorrheal effect at all concentrations and not showing spasmolytic effect (Araújo et al. 2024).

## Conclusion

This review highlights the bioactive compounds and medicinal potential of *Cannabis sativa* roots, broadening current understanding of this historically important yet scientifically underexplored plant organ. While *C. sativa* has long been recognized as a medicinal species, research has largely focused on the psychoactive properties of its aerial parts, overshadowing the pharmacological relevance of its roots.

Evidence to date demonstrates that *C. sativa* roots possess antioxidant, anti-inflammatory, antinociceptive, and antispasmodic activities, as well as promising antibacterial and antifungal properties, with no reports of cytotoxicity or genotoxicity. Their rich content of secondary metabolites, including terpenes, phytosterols, phenolic compounds, alkaloids, and cannabinoids, positions the roots as a valuable source of pharmacologically active molecules with potential therapeutic applications.

### Strengths, limitations, and future prospects

This review contributes by integrating scattered evidence on the phytochemistry and pharmacological activities of *C. sativa* roots, providing a comprehensive overview rather than isolated discussions of extraction techniques or single therapeutic uses. However, the heterogeneity of geographical origins and environmental conditions across the reviewed studies represents a major limitation, as factors such as climate, soil composition, and harvest time strongly influence phytochemical profiles and complicate comparisons. Despite these gaps, the findings underscore that systematic and standardized studies on *C. sativa* roots may yield novel bioactive compounds with significant pharmacological value.

Future research should prioritize detailed phytochemical characterization, mechanistic pharmacological studies, and controlled in vivo and clinical evaluations. Ultimately, the roots of *C. sativa* represent a neglected pharmacognostic resource with considerable potential to contribute to drug discovery and the development of new therapeutic strategies.

## Data Availability

No datasets were generated or analysed during the current study.

## References

[CR1] Abdel-Sattar E, Abou-Hussein D, Petereit F. Chemical constituents from the leaves of *Euphorbia ammak* growing in Saudi Arabia. Pharmacogn Res. 2015;7(1):14. 10.4103/0974-8490.147136. PMC4285643 25598629

[CR2] Alam S, Richi FT, Emon NU, Chowdhury AA, Hasan CM, Haque MR. First- time report on compound isolation from two Colocasia species: vegetable- derived bioactive metabolites and their medicinal potential. Front Pharmacol. 2024;15. 10.3389/fphar.2024.1474706. PMC11652197 39697535

[CR3] Alavi M, Ashengroph M. Mycosynthesis of AgNPs: mechanisms of nanoparticle formation and antimicrobial activities. Expert Rev Anti- Infect Ther. 2023;21:355–63. 10.1080/14787210.2023.2179988. Taylor & Francis. 36786717

[CR4] Alloatti A, Tripodi KEJ, Uttaro AD. Synergistic effect of inhibitors of fatty acid desaturases on Trypanosoma parasites. Parasitol Res. 2013;112(9):3289–94. 10.1007/s00436-013-3508-y. 23797276

[CR5] Angsusing J, Singh S, Samee W, Tadtong S, Stokes L, O'Connell M, et al. Anti- Inflammatory Activities of Yataprasen Thai Traditional Formulary and Its Active Compounds, Beta-Amyrin and Stigmasterol, in RAW264.7 and THP-1 Cells. Pharmaceuticals (Basel). 2024;17(8). 10.3390/ph17081018. PMC11357128 39204123

[CR6] Araújo TCdeL, Menezes PMN, Ribeiro TF, Macêdo CAF, Souza NACde, Lima KSB, et al. *Cannabis sativa* L. roots from Northeast Brazil reduce abdominal contortions in a mouse model of primary dysmenorrhea. J Ethnopharmacol. 2024;318:116891. 10.1016/j.jep.2023.116891. 37423518

[CR7] Arya SS, Rookes JE, Cahill DM, Lenka SK. Vanillin: a review on the therapeutic prospects of a popular flavouring molecule. Adv Trad Med. 2021;21:1–17. 10.1007/s13596-020-00531-w. Springer.

[CR8] Bakrim S, Elouafy Y, Touhtouh J, Aanniz T, El Kadri K, Khalid A, et al. Exploring the chemistry, biological effects, and mechanism insights of natural coumaroyltyramine: First report. Phytotherapy. 2024;178:106182. 10.1016/j.fitote.2024.106182. Fitoterapia. 39153554

[CR9] Baptista F, Paié-Ribeiro J, Almeida M, Barros AN. Exploring the Role of Phenolic Compounds in Chronic Kidney Disease: A Systematic Review. Molecules. 2024;29:2576. 10.3390/molecules29112576. Multidisciplinary Digital Publishing Institute. PMC11173950 38893451

[CR10] Barkas F, Bathrellou E, Nomikos T, Panagiotakos D, Liberopoulos E, Kontogianni MD. Plant Sterols and Plant Stanols in Cholesterol Management and Cardiovascular Prevention. Nutrients. 2023;15. 10.3390/nu15132845. Multidisciplinary Digital Publishing Institute (MDPI). PMC10343346 37447172

[CR11] Barrales-Cureño HJ, López-Valdez LG, Reyes C, Cetina-Alcalá VM, Vasquez- García I, Diaz-Lira OF, et al. Chemical characteristics, therapeutic uses, and legal aspects of the cannabinoids of Cannabis sativa: A review. Brazilian Arch Biol Technol. 2020;63:e20190222. 10.1590/1678-4324-2020190222. Paraná Institute of Technology - Tecpar.

[CR12] Bartel I, Mandryk I, Horbańczuk JO, Wierzbicka A, Koszarska M. Nutraceutical Properties of Syringic Acid in Civilization Diseases-Review. Nutrients. 2024;16. 10.3390/nu16010010. PMC10780450 38201840

[CR13] Belén Valdez M, D′Jonsiles MF, Avigliano E, Palermo JA. Unlocking the Potential of Glutinol: Structural Diversification and Antifungal Activity against Phytopathogenic Fusarium Strains. J Nat Prod. 2024 ; 10.1021/acs.jnatprod.4c00566. 39101318

[CR14] Chen HH, Chen YT, Huang YW, Tsai HJ, Kuo CC. 4-Ketopinoresinol, a novel naturally occurring ARE activator, induces the Nrf2/HO-1 axis and protects against oxidative stress-induced cell injury via activation of PI3K/AKT signaling. Free Radic Biol Med. 2012;52(6):1054–66. 10.1016/j.freeradbiomed.2011.12.012. 22245092

[CR15] Chiou WC, Lyu YS, Hsia TL, Chen JC, Lin LC, Chang MF, et al. Ergosterol peroxide blocks HDV infection as a novel entry inhibitor by targeting human NTCP receptor. Biomed Pharmacother. 2024;1:170. 10.1016/j.biopha.2023.116077. 38154274

[CR16] Chua RW, Song KP, Ting ASY. Characterization and identification of antimicrobial compounds from endophytic Fusarium incarnatum isolated from Cymbidium orchids. Int Microbiol. 2024;27(4):977–92. 10.1007/s10123-023-00442-1. 37975992

[CR17] Dalli M, Azizi SE, Azghar A, Saddari A, Benaissa E, Lahlou YB, et al. Cannabis sativa L.: A comprehensive review on legislation, decriminalization, phytochemistry, antimicrobial activity, and safety. J Food Drug Anal. 2023;31(3):408–35. 10.38212/2224-6614.3471. PMC10629910 39666278

[CR18] de Carvalho JF, Ribeiro MFLDS, Skare T. Cannabis therapy in rheumatological diseases: A systematic review. Northern Clin Istanbul. 2024;11:361–6. 10.14744/nci.2023.43669. PMC11331211 39165706

[CR19] de Castro TN, Mota MD, Cazedey ECL. Photoprotective and antioxidant activity of phenolic compounds: a systematic review of in vitro tests. Rev Colomb Ciencias Quim. 2022;51(2):557–88. 10.15446/rcciquifa.v51n2.97604.

[CR20] de Miranda RS, de Jesus BSM, da Silva Luiz SR, Viana CB, Adão Malafaia CR, Figueiredo FS, et al. Antiinflammatory activity of natural triterpenes-An overview from 2006 to 2021. Phytother Res. 2022;36:1459–506. 10.1002/ptr.7359. 35229374

[CR21] Deng S, Liu Y, Liu X, Yu J, Chen Y, Huo J. Inhibition of colorectal cancer aggressiveness by Oleanolic acid through Nur77 degradation. Phytomedicine. 2024;1:135. 10.1016/j.phymed.2024.156192. 39520953

[CR22] Duan C, Liu Y, Hao Z, Wang J. Ergosterol peroxide suppresses porcine deltacoronavirus (PDCoV)-induced autophagy to inhibit virus replication via p38 signaling pathway. Vet Microbiol. 2021;1:257. 10.1016/j.vetmic.2021.109068. PMC8035807 33894664

[CR23] Duarte N, Ferreira MJU, Martins M, Viveiros M, Amaral L. Antibacterial activity of ergosterol peroxide against *Mycobacterium tuberculosis*: dependence upon system and medium employed. Phyther Res. 2007;21(7):601–4. 10.1002/ptr.2119. 17357175

[CR24] Eguchi R, Ono N, Hirai Morita A, Katsuragi T, Nakamura S, Huang M, et al. Classification of alkaloids according to the starting substances of their biosynthetic pathways using graph convolutional neural networks. BMC Bioinformatics. 2019;20(1). 10.1186/s12859-019-2963-6. PMC6617615 31288752

[CR25] Elhendawy MA, Wanas AS, Radwan MM, Azzaz NA, Toson ES, Elsohly MA. Chemical and Biological Studies of Cannabis sativa Roots. Med Cannabis Cannabinoids. 2019;1(2):104–11. 10.1159/000495582. PMC7158750 32296742

[CR26] Fantoukh OI, Al-Hamoud GA, Nasr FA, Almarfadi OM, Hawwal MF, Ali Z, et al. Revisiting the Flora of Saudi Arabia: Phytochemical and Biological Investigation of the Endangered Plant Species Euphorbia saudiarabica. Metabolites. 2023;13(4):556. 10.3390/metabo13040556. PMC10144502 37110214

[CR27] Fernandes A, Rodrigues PM, Pintado M, Tavaria FK. A systematic review of natural products for skin applications: Targeting inflammation, wound healing, and photo-aging. Phytomedicine. 2023;115:154824. 10.1016/j.phymed.2023.154824. Urban & Fischer. 37119762

[CR28] Ferrini F, Donati Zeppa S, Fraternale D, Carrabs V, Annibalini G, Verardo G, et al. Characterization of the Biological Activity of the Ethanolic Extract from the Roots of Cannabis sativa L. Grown Aeroponics Antioxidants. 2022;11(5):860. 10.3390/antiox11050860. PMC9137677 35624724

[CR29] Freitas da Silva FE, das Chagas Lima Pinto F, Loiola Pessoa OD, Marques da Fonseca A, Martins da Costa JG, Pinheiro Santiago GM. Campesterol semi- synthetic derivatives as potential antibacterial: in vitro and in silico evaluation. Chem Biodivers. 2023. 10.1002/cbdv.202300536. 37335297

[CR30] Gagné V, Merindol N, Boucher R, Boucher N, Desgagné-Penix I. Rooted in therapeutics: comprehensive analyses of *Cannabis sativa* root extracts reveals potent antioxidant, anti-inflammatory, and bactericidal properties. Front Pharmacol. 2024;15:1465136. 10.3389/fphar.2024.1465136. PMC11440120 39351095

[CR31] Galdiero E, Ricciardelli A, D’Angelo C, de Alteriis E, Maione A, Albarano L, et al. Pentadecanoic acid against Candida albicans-Klebsiella pneumoniae biofilm: towards the development of an anti-biofilm coating to prevent polymicrobial infections. Res Microbiol. 2021;172(7-8). 10.1016/j.resmic.2021.103880. 34563667

[CR32] Gu Z, Singh S, Niyogi RG, Lamont GJ, Wang H, Lamont RJ, et al. Marijuana- Derived Cannabinoids Trigger a CB2/PI3K Axis of Suppression of the Innate Response to Oral Pathogens. Front Immunol. 2019;15(10):427103. 10.3389/fimmu.2019.02288. PMC6804395 31681262

[CR33] Haghdoost M, Peters EN, Roberts M, Bonn-Miller MO. Tetrahydrocannabivarin is Not Tetrahydrocannabinol. Cannabis Cannabinoid Res. 2024. 10.1089/can.2024.005. PMC11947634 38995871

[CR34] Han L, Ren J, Xue Y, Xie G, Gao J, Fu Q, et al. Palmitoleic acid inhibits Pseudomonas aeruginosa quorum sensing activation and protects lungs from infectious injury. Respir Res. 2024;25(1). 10.1186/s12931-024-03035-2. PMC11613874 39623416

[CR35] Huang J, Yi L, Yang X, Zheng Q, Zhong J, Ye S, et al. Neural stem cells transplantation combined with ethyl stearate improves PD rats motor behavior by promoting NSCs migration and differentiation. CNS Neurosci Ther. 2023;29(6):1571–84. 10.1111/cns.14119. PMC10173712 36924304

[CR36] Huang S, Li H, Xu J, Zhou H, Seeram NP, Ma H, et al. Chemical constituents of industrial hemp roots and their anti-inflammatory activities. J Cannabis Res. 2023;5(1):1–8. 10.1186/s42238-022-00168-3. PMC9841654 36642726

[CR37] Hurgobin B, Tamiru-Oli M, Welling MT, Doblin MS, Bacic A, Whelan J, et al. Recent advances in Cannabis sativa genomics research. New Phytol. 2021;230:73–89. 10.1111/nph.17140. John Wiley & Sons, Ltd. PMC7986631 33283274

[CR38] Intisar A, Zhang L, Luo H, Kiazolu JB, Zhang R, Zhang W. Anticancer Constituents and Cytotoxic Activity of Methanol-Water Extract of Polygonum Bistorta L. African J Tradit Complement Altern Med. 2012;10(1):53. 10.4314/ajtcam.v10i1.9. PMC3746358 24082326

[CR39] Jiang, H., Li, J., Chen, A., Xia, M., Guo, P., Yao, S., Chen, S. [Retracted] Fucosterol exhibits selective antitumor anticancer activity against HeLa human cervical cell line by inducing mitochondrial mediated apoptosis, cell cycle migration inhibition and downregulation of m-TOR/PI3K/Akt signalling pathway. Oncol Lett 2023: 32 10.3892/ol.2022.13618. PMC9773325 36589662

[CR40] Jelenko C, Wheeler ML, Anderson AP, Callaway BD, McKinley JC. Studies in burns: XIV. Healing in burn wounds treated with ethyl linoleate alone or in combination with selected topical antibacterial agents. Ann Surg. 1975;182(5):562–6. 10.1097/00000658-197511000-00005. PMC1344038 1190861

[CR41] Judžentienė A, Garjonytė R, Būdienė J. Phytochemical composition and antioxidant activity of various extracts of fibre hemp (*Cannabis sativa* L.) cultivated in Lithuania. Molecules. 2023;28(13):4928. 10.3390/molecules28134928. PMC10343532 37446590

[CR42] Khan Z, Nath N, Rauf A, Emran TB, Mitra S, Islam F, et al. Multifunctional roles and pharmacological potential of β-sitosterol: Emerging evidence toward clinical applications. Chemico-Biol Interact. 2022;365:110117. 10.1016/j.cbi.2022.110117. 35995256

[CR43] Komatsu Y, Takehara M, Hart X, Takahashi Y, Hori S, Ueno F, et al. Advancements in Non-Dopaminergic Treatments for Schizophrenia: A Systematic Review of Pipeline Developments. Pharmacopsychiatry. Georg Thieme Verlag; 2024. 10.13039/501100001691. 38710208

[CR44] Kornpointner C, Sainz Martinez A, Marinovic S, Haselmair-Gosch C, Jamnik P, Schröder K, et al. Chemical composition and antioxidant potential of Cannabis sativa L. roots. Ind Crops Prod. 2021;1(165):113422. 10.1016/j.indcrop.2021.113422.

[CR45] Li X, Lin B, Lin Z, Ma Y, Wang Q, Zheng Y, et al. Exploration in the mechanism of fucosterol for the treatment of non-small cell lung cancer based on network pharmacology and molecular docking. Sci Rep. 2021;11(1). 10.1038/s41598-021-84380-w PMC7921686 33649481

[CR46] Liu Y, Wang X, Wang J, Zhang J, Duan C, Wang J. Ergosterol peroxide inhibits porcine epidemic diarrhea virus infection in Vero cells by suppressing ROS generation and p53 activation. Viruses. 2022. 10.3390/v14020402. PMC8876155 35215995

[CR47] Liu J, Ma R, Fu B, Yang P, Zhang L, Zhang C, et al. Phytosterols in mountaincultivated ginseng, as master healthy aging dietary supplement, activates steroid signaling in aging Drosophila melanogaster. Exp Gerontol. 2024;195:112554. 10.1016/j.exger.2024.112554. 39179161

[CR48] Londero VS, da Costa-Silva TA, Gomes KS, Ferreira DD, Mesquita JT, Tempone AG, et al. Acetylenic fatty acids from *Porcelia macrocarpa* (Annonaceae) against trypomastigotes of *Trypanosoma cruzi*: effect of octadec- 9-ynoic acid in plasma membrane electric potential. Bioorg Chem. 2018;78:307–11. 10.1016/j.bioorg.2018.03.025. 29625270

[CR49] Lowe H, Steele B, Bryant J, Toyang N, Ngwa W. Non-cannabinoid metabolites of cannabis sativa l With therapeutic potential. Plants. 2021;10(2):1–17. 10.3390/plants10020400. PMC7923270 33672441

[CR50] Lukman HY, Kuo Y, Owolabi MS, Lawal B, Chen LC, Ajenifujah OT, et al. Evaluation of terpenes rich *Hura crepitans* extract on glucose regulation and diabetic complications in STZ-induced diabetic rats. Biomed Pharmacother. 2024;179:117308. 10.1016/j.biopha.2024.117308. 39180791

[CR51] Maiocchi A, Fumagalli M, Vismara M, Blanco A, Ciriello U, Paladino G, et al. Minor Cannabinoids as Inhibitors of Skin Inflammation: Chemical Synthesis and Biological Evaluation. J Nat Prod. 2024;87(7):1725–34. 10.1021/acs.jnatprod.4c00212. 38889235

[CR52] Masi M, Koirala M, Delicato A, Di Lecce R, Merindol N, Ka S, et al. Isolation and biological characterization of homoisoflavanoids and the alkylamide n-p- coumaroyltyramine from crinum biflorum rottb., an amaryllidaceae species collected in Senegal. Biomolecules. 2021;11(9). 10.3390/biom11091298. PMC8466962 34572511

[CR53] Mazzarotti G, Cuomo M, Ragosta MC, Russo A, D’Angelo M, Medugno A, et al. Oleanolic Acid Modulates DNA Damage Response to Camptothecin Increasing Cancer Cell Death. Int J Mol Sci. 2024;25(24):13475. 10.3390/ijms252413475. PMC11676975 39769237

[CR54] Menezes PMN, de Araújo TCL, Pereira ECV, Neto JA, Silva DS, Brito MC, et al. Investigation of antinociceptive, antipyretic, antiasthmatic, and spasmolytic activities of Brazilian Cannabis sativa L roots in rodents. J Ethnopharmacol. 2021;278:114259. 10.1016/j.jep.2021.114259. 34058314

[CR55] Miya GM, Oriola AO, Payne B, Cuyler M, Lall N, Oyedeji AO. Steroids and Fatty Acid Esters from Cyperus sexangularis Leaf and Their Antioxidant, Anti- Inflammatory and Anti-Elastase Properties. Molecules. 2023;28(8):3434. 10.3390/molecules28083434. PMC10141076 37110668

[CR56] Mo W, Wang C, Li J, Chen K, Xia Y, Li S, et al. Fucosterol protects against concanavalin a-induced acute liver injury: Focus on P38 MAPK/NF-κ B pathway activity. Gastroenterol Res Pract. 2018;2018:2824139. 10.1155/2018/2824139. PMC6079550 30116260

[CR57] Moges GW, Manahelohe GM, Assege MA, Sewachen Tasew B, Molla DK, Belew AA, et al. Phytochemical profiles and biological activity of Myrsine africana L.: a comprehensive review. Front Pharmacol. 2025;16:1565656. 10.3389/fphar.2025.1565656. PMC11965677 40183106

[CR58] Odieka AE, Obuzor GU, Oyedeji OO, Gondwe M, Hosu YS, Oyedeji AO. The Medicinal Natural Products of Cannabis sativa Linn.: A Review. Molecules. 2022;27:1689. 10.3390/molecules27051689. Multidisciplinary Digital Publishing Institute. PMC8911748 35268790

[CR59] Oliveira LR, Vidal DM, Freitas TR, de P. Sabino A, Duarte LP, de Sousa GF. Synthesis and Cytotoxic Activity of Friedelinyl Esters. Chem Biodivers. 2024;21(9):e202400652. 10.1002/cbdv.202400652. 38924342

[CR60] Omar RMK, Igoli J, Gray AI, Ebiloma GU, Clements C, Fearnley J, et al. Chemical characterization of Nigerian red propolis and its biological activity against *Trypanosoma Brucei*. Phytochem Anal. 2016;27(2):107–15. 10.1002/pca.2605. 26662866

[CR61] Oreopoulou A, Choulitoudi E, Tsimogiannis D, Oreopoulou V. Six common herbs with distinctive bioactive, antioxidant components. A review of their separation techniques. Molecules. 2021;26:2920. 10.3390/molecules26102920. Multidisciplinary Digital Publishing Institute. PMC8157015 34069026

[CR62] Park SY, Seetharaman R, Ko MJ, Kim DY, Kim TH, Yoon MK, et al. Ethyl linoleate from garlic attenuates lipopolysaccharide-induced pro-inflammatory cytokine production by inducing heme oxygenase-1 in RAW264.7 cells. Int Immunopharmacol. 2014;19(2):253–61. 10.1016/j.intimp.2014.01.017. 24508058

[CR63] Pamunuwa G, Karunaratne DN, Waisundara VY. Antidiabetic Properties, Bioactive Constituents, and Other Therapeutic Effects of Scoparia dulcis. Evid Based Complement Alternat Med. 2016;2016. 10.1155/2016/8243215 PMC4995349 27594892

[CR64] Pathak S, Jeyabalan JB, Liu K, Cook P, Lange B, Kim S, et al. Assessing effects of Cannabis on various neuropathologies: A systematic review. J Ayurveda Integr Med. 2024;15:100911. 10.1016/j.jaim.2024.100911. Elsevier B.V.. PMC11282377 38876946

[CR65] Pellati F, Borgonetti V, Brighenti V, Biagi M, Benvenuti S, Corsi L. Cannabis sativa L. and Nonpsychoactive Cannabinoids: Their Chemistry and Role against Oxidative Stress, Inflammation, and Cancer. BioMed Res Int. 2018;2018:1691428. 10.1155/2018/1691428. Hindawi Limited. PMC6304621 30627539

[CR66] Perumal P, Sowmiya R, Prasanna Kumar S, Ravikumar S, Deepak P, Balasubramani G. Isolation, structural elucidation and antiplasmodial activity of fucosterol compound from brown seaweed, Sargassum linearifolium against malarial parasite Plasmodium falciparum. Nat Prod Res. 2018;32(11):1316–9. 10.1080/14786419.2017.1342081. 28637390

[CR67] Purwantiningsih TI, Widyobroto BP, Suranindyah YY, Artama WT. Antibacterial activity of faloak (Sterculia quadrifida R Br) bark: a promising natural antibacterial candidate. Farmacia. 2024;72(4):832–9. 10.31925/farmacia.2024.4.11.

[CR68] Radwan MM, Chandra S, Gul S, Elsohly MA. Cannabinoids, phenolics, terpenes and alkaloids of cannabis. Molecules. 2021;26:2774. 10.3390/molecules26092774. PMC8125862 34066753

[CR69] Roohi TF, Krishna KL, Shakeel F. Synergistic modulation of endoplasmic reticulum stress pathway, oxidative DNA damage and apoptosis by β-amyrin and metformin in mitigating hyperglycemia-induced renal damage using adult zebrafish model. BMC Pharmacol Toxicol. 2024;25(1):66. 10.1186/s40360-024-00797-9. PMC11430224 39334288

[CR70] Roy A, Kundu M, Chakrabarti S, Patel DR, Pahan K. Oleamide, a Sleep- Inducing Supplement, Upregulates Doublecortin in Hippocampal Progenitor Cells via PPARα. J Alzheimer’s Dis. 2021;84(4):1747–62. 10.3233/JAD-21512. PMC10075226 34744082

[CR71] Ryz NR, Remillard DJ, Russo EB. Cannabis roots: a traditional therapy with future potential for treating inflammation and pain. Cannabis Cannabinoid Res. 2017;2:210–6. 10.1089/can.2017.0028. PMC5628559 29082318

[CR72] Saeed NM, El-Demerdash E, Abdel-Rahman HM, Algandaby MM, Al-Abbasi FA, Abdel-Naim AB. Anti-inflammatory activity of methyl palmitate and ethyl palmitate in different experimental rat models. Toxicol Appl Pharmacol. 2012;264(1):84–93. 10.1016/j.taap.2012.07.020. 22842335

[CR73] Sagna A, Nair RVR, Hulyalkar N, Rajasekharan S, Nair VTG, Sivakumar KC, et al. Ethyl palmitate, an anti-chikungunya virus principle from Sauropus androgynus, a medicinal plant used to alleviate fever in ethnomedicine. J Ethnopharmacol. 2023;12:309. 10.1016/j.jep.2023.116366. 36914036

[CR74] Saini V, Mata Espinosa D, Pandey A, Dighe V, Barrios Payán J, Prasad Myneedu V, et al. Antimycobacterial Activity of Solid Lipid Microparticles Loaded with Ursolic Acid and Oleanolic Acid: In Vitro, In Vivo, and Toxicity Assessments. Microorganisms. 2024;12(11). 10.3390/microorganisms12112140. PMC11596724 39597530

[CR75] Saleska JL, Bryant C, Kolobaric A, D’Adamo CR, Colwell CS, Loewy D, et al. The Safety and Comparative Effectiveness of Non-Psychoactive Cannabinoid Formulations for the Improvement of Sleep: A Double-Blinded, Randomized Controlled Trial. J Am Nutr Assoc. 2024;43(1):1–11. 10.1080/27697061.2023.2203221. 37162192

[CR76] Schilling S, Melzer R, McCabe PF. Cannabis sativa. Curr Biol. 2020;30:R8-9 http://www.cell.com/article/S096098221931379X/fulltext. Cell Press. 10.1016/j.cub.2019.10.039 31910378

[CR77] Shokry S, Hegazy A, Abbas AM, Mostafa I, Eissa IH, Metwaly AM, et al. Phytoestrogen β-Sitosterol Exhibits Potent In Vitro Antiviral Activity against Influenza A Viruses. Vaccines. 2023;11(2). 10.3390/vaccines11020228. PMC9964242 36851106

[CR78] Singh SK, Shrivastava S, Mishra AK, Kumar D, Pandey VK, Srivastava P, et al. Friedelin: Structure, Biosynthesis, Extraction, and Its Potential Health Impact. Molecules. 2023;28:7760. 10.3390/molecules28237760. Multidisciplinary Digital Publishing Institute (MDPI). PMC10707989 38067489

[CR79] Suman S, Loveleen L, Bhandari M, Syed A, Bahkali AH, Manchanda R, et al. Antibacterial, antioxidant, and haemolytic potential of silver nanoparticles biosynthesized using roots extract of Cannabis sativa plant. Artif Cells Nanomed Biotechnol. 2022;50(1):343–51. 10.1080/21691401.2022.2149543. 36519372

[CR80] Tabashsum Z, Angeles-Solano M, Sidders AE, Parsons JB, Rowe SE. Palmitoleic acid sensitizes vancomycin-resistant Staphylococcus aureus to vancomycin by outpacing the expression of resistance genes. Ruzzini A, editor. Microbiol Spectr. 2025;13(1). 10.1128/spectrum.01996-24. PMC11705785 39656010

[CR81] Taher MA, Hossain MJ, Zahan MS, Hasan MM, Ferdous J, Rahman A, et al. Phyto-pharmacological and computational profiling of *Bombax ceiba* Linn. leaves revealed pharmacological properties against oxidation, hyperglycemia, pain, and diarrhea. Heliyon. 2024;10(15):e35422. 10.1016/j.heliyon.2024.e35422. PMC11336592 39170236

[CR82] Venn-Watson S, Schork NJ. Pentadecanoic Acid (C15:0), an Essential Fatty Acid, Shares Clinically Relevant Cell-Based Activities with Leading Longevity- Enhancing Compounds. Nutrients. 2023;15(21):4607. 10.3390/nu15214607. PMC10649853 37960259

[CR83] Waghole RJ, Misar AV, Kulkarni NS, Khan F, Naik DG, Jadhav SH. In vitro and in vivo anti-inflammatory activity of Tetrastigma sulcatum leaf extract, pure compound and its derivatives. Inflammopharmacology. 2022;30(1):291–311. 10.1007/s10787-021-00897-6. 35064430

[CR84] Wang R, Deng X, Gao Q, Wu X, Han L, Gao X, et al. Sophora alopecuroides L.: An ethnopharmacological, phytochemical, and pharmacological review. J Ethnopharmacol. 2020;2481:12172. 10.1016/j.jep.2019.112172. 31442619

[CR85] Wang X, Zhu H, Liu T, Guo Z, Zhao C, He Z, et al. Comparison of various doses of oral cannabidiol for treating refractory epilepsy indications: a network meta-analysis. Front Neurol. 2024;15:1243597. 10.3389/fneur.2024.1243597. PMC11238246 38994494

[CR86] Whynot EG, Tomko AM, Dupré DJ. Anticancer properties of cannabidiol and Δ9-tetrahydrocannabinol and synergistic effects with gemcitabine and cisplatin in bladder cancer cell lines. J Cannabis Res. 2023;5(1). 10.1186/s42238-023-00174-z. PMC9985258 36870996

[CR87] Xu Z, Sun H, Wang Z, Li J, Gu J. Identifying biomarkers for nasopharyngeal carcinoma diagnosis and chemoradiotherapy response using serum endogenous small molecules profiling. J Pharm Biomed Anal. 2024;251:116428. 10.1016/j.jpba.2024.116428. 39208647

[CR88] Xu X, He Z, Zhang Q, Lu Q, Zhu M, Zu Y, et al. Constituents, antioxidant and antibacterial abilities of essential oil of *Dolichos lablab* flowers and its application as food preservative. Flavour Fragr J. 2025;40:537–54. 10.1002/ffj.3843.

[CR89] Yan Y, Li X, Zhang C, Lv L, Gao B, Li M. Research progress on antibacterial activities and mechanisms of natural alkaloids: A review. Antibiotics. 2021;10:318. 10.3390/antibiotics10030318. Multidisciplinary Digital Publishing Institute. PMC8003525 33808601

[CR90] Yu C, Nian Y, Chen H, Liang S, Sun M, Pei Y, et al. Pyranone Derivatives With Antitumor Activities, From the Endophytic Fungus Phoma sp. YN02-P-3. Front Chem. 2022;10:950726. 10.3389/fchem.2022.950726. PMC9300907 35873041

[CR91] Yu GR, Jin HL, Kim DH, Noah JK, Kim H, Lim DW, et al. A comparative study on enhanced anti-inflammatory activity of Cannabis root extract prepared by combined processing process. J Korean Med. 2024;45(2):41–54. 10.13048/jkm.24023.

[CR92] Yuan Q, Xie F, Huang W, Hu M, Yan Q, Chen Z, et al. The review of alpha- linolenic acid: Sources, metabolism, and pharmacology. Phytother Res. 2022;36:164–88. 10.1002/ptr.7295. 34553434

[CR93] Zeng LR, Pan BW, Cai J, Liu LJ, Dong ZC, Zhou Y, et al. Construction, structural modification, and bioactivity evaluation of pentacyclic triterpenoid privileged scaffolds in active natural products. RSC Adv. 2024;14(53):39436–61. 10.1039/d4ra07602h. PMC11640457 39679419

[CR94] Zhou B, Liang X, Feng Q, Li J, Pan X, Xie P, et al. Ergosterol peroxide suppresses influenza A virus-induced pro-inflammatory response and apoptosis by blocking RIG-I signaling. Eur J Pharmacol. 2019;5:860. 10.1016/j.ejphar.2019.172543. 31323223

[CR95] Zhou Y, Lin S, Zhong X, Huang F, Huang J, Xu L. Oleanolic acid combined with aspirin plays antitumor roles in colorectal cancer via the Akt/NFκB/IκBα/COX2 pathway. Cell death Discov. 2024;10(1). 10.1038/s41420-024-02223-9. PMC11655652 39695129

[CR96] Zhu S, Jiao W, Xu Y, Hou L, Li H, Shao J, et al. Palmitic acid inhibits prostate cancer cell proliferation and metastasis by suppressing the PI3K/Akt pathway. Life Sci. 2021;286:120046. 10.1016/j.lfs.2021.120046. 34653428

